# Differential Atrophy in the Hippocampal Subfield Volumes in Four Types of Mild Dementia

**DOI:** 10.3389/fnins.2020.00699

**Published:** 2020-07-09

**Authors:** Lin Huang, Keliang Chen, Xiaochen Hu, Qihao Guo

**Affiliations:** ^1^Department of Gerontology, Shanghai Jiao Tong University Affiliated Sixth People’s Hospital, Shanghai, China; ^2^Department of Neurology, Huashan Hospital, Shanghai Medical College, Fudan University, Shanghai, China; ^3^Department of Psychiatry and Psychotherapy, Medical Faculty, University of Cologne, Cologne, Germany

**Keywords:** Alzheimer’s disease, dementia with Lewy bodies, semantic dementia, posterior cortical atrophy, volumetric MRI

## Abstract

**Objectives:**

To investigate the bilateral hippocampal subfield volumetric differences in four types of mild dementia, namely typical Alzheimer’s disease (tAD), dementia with Lewy bodies (DLB), semantic dementia (SD), and posterior cortical atrophy (PCA), to assist differential diagnosis.

**Methods:**

One hundred three participants, including 22 tAD, 34 SD (17 left SD and 17 right SD), 15 DLB, 12 PCA patients, and 20 normal controls (NC), were recruited. All subjects received standard neuropsychological assessments and magnetic resonance imaging (MRI). The hippocampal subfields were automatically segmented via Freesurfer. The study compared the volumetric differences and used the receiver operating characteristic (ROC) curves to estimate the efficacy of each hippocampal subfield to distinguish between groups. Spearman correlation analysis was used to investigate the relationship between memory recall scores and hippocampal subfield volumes.

**Results:**

The hippocampal subfield atrophy varied in different groups: tAD, SD, and PCA patients had subregional atrophy in bilateral hippocampi compared to NC, and DLB patients showed preserved volumes; left SD patients suffered the most severe atrophy of the left hippocampus, and right SD patients were atrophied mostly in the right hippocampus. There was no significant difference in the volume of hippocampal subregions between tAD and PCA subjects, but the former tended to be atrophied more asymmetrically. ROC analysis showed that, for discrimination, the areas under the curve (AUC) of some subfields were larger than the total hippocampus, but none observed significant difference. In addition, immediate recall scores were correlated to left CA1, CA2/3, CA4/DG, subiculum, and presubiculum (*p* < 0.05), and delayed recall scores were strongly related to bilateral CA2/3, CA4/DG, subiculum, and presubiculum (*r* = 0.38–0.52, *p* < 0.05).

**Conclusion:**

Differential atrophy patterns in the bilateral hippocampal subfield volumes could serve the differential diagnosis in patients with different causes of mild dementia: left CA1 for tAD; left presubiculum for LSD; right CA4/DG, right presubiculum, and right subiculum for RSD; CA4/DG and right CA2/3 for DLB; right CA2/3 and right CA4/DG for PCA. Additionally, several hippocampal subfield volumes were significantly associated with memory scores, further highlighting the essential role of the hippocampus in memory decline.

## Introduction

Structural magnetic resonance imaging (MRI) and volumetric studies can be helpful in the differential diagnosis by demonstrating focal changes in the brain volumes with different causes of dementia. In typical Alzheimer’s disease (tAD), the hippocampus, known to play an essential role in the consolidation of information from short- to long-term memory, is one of the earliest structures that are vulnerable to atrophy and, thus, has been recognized as the core biomarker for the progression from mild cognitive impairment (MCI) to dementia (Mu and Gage, 2011).

Recently, mounting evidence suggests that the decrease in the total volume of the hippocampus is no longer specific for AD since it can exist in non-AD forms of dementia and may be more suitable for the disease course monitoring (Hatanpaa et al., 2014). The hippocampus consists of several anatomically and functionally diverse subfields, including the dentate gyrus (DG), the cornu ammonis (CA) areas 1–4, the subiculum, and the presubiculum (Freundl and Buzsi, 2015). These subfields might be selectively damaged in patients with different causes of dementia. A recent study comparing 30 AD patients, 41 MCI patients, and 38 healthy controls with an automated segmentation protocol for the volumetric analysis of hippocampal subfields reports a prevalent atrophy of the presubicular-subicular complex from the early phases of AD (Carlesimo et al., 2015). However, given the complexity of the internal structure of the hippocampus, the different patterns of hippocampal subfield atrophy in different dementias has not been clarified.

Dementia with Lewy bodies (DLB) is accompanied by changes in behavior, cognition, and movement. Persistent memory impairment may not necessarily occur in the early stages but is usually evident with progression (Calderon et al., 2001). Previous studies show that DLB had significant decline in CA1 and subiculum compared to NC (Chow et al., 2012) but less atrophy of CA1 and subiculum in comparison to AD (Firbank et al., 2010; Mak et al., 2016).

Semantic dementia (SD) is characterized by loss of semantic memory and relatively intact episodic memory compared to AD (Gorno-Tempini et al., 2011). SD is often associated with predominant anterior temporal lobe atrophy, manifested as asymmetrical atrophy of the left or right cerebral hemispheres, and can be further classified as left SD (LSD) and right SD (RSD) (Gorno-Tempini et al., 2011; Chen et al., 2018). One study reports a severe neuronal loss in the CA1 subfield in SD patients although the DG subfield is relatively spared (La Joie et al., 2013).

Posterior cortical atrophy (PCA) is characterized by a progressive decline in visual processing skills and other functions subserved by parietal, occipital, and occipitotemporal regions (Crutch et al., 2012). Although episodic memory is relatively spared in the early stages, the hippocampal volume is still significantly decreased compared with the normal controls (NC), especially in the superior hippocampal tail (Manning et al., 2015).

In this study, we intended to compare the different patterns of hippocampal subfield atrophy among tAD, DLB, SD, and PCA patients based on volumetric MRI measurements. We conducted segmentation of the hippocampal subfields via an automated approach and expected to identify hippocampal subfields that could distinguish between groups.

## Materials and Methods

### Participants

A total of 103 subjects were recruited in the present study, including 22 tAD patients, 34 SD patients (17 LSD and 17 RSD patients), 15 DLB patients, 12 PCA patients, and 20 NC. Patients were recruited from the memory clinic of Huashan Hospital in Shanghai, China, from August 2016 to October 2018. NC subjects were randomly selected from their relatives or friends during the same period. All subjects underwent a general neurological examination, a comprehensive neuropsychological assessment, and brain MRI. This study was approved by the ethics committee of Huashan Hospital, and all subjects provided written consent.

Inclusion criteria were aged 40 to 80 years; at least 7 years of education; and no history of alcoholism, drug abuse, head trauma, or psychiatric disorders. The diagnosis of all patients was made on clinical grounds and confirmed by two experienced neurologists according to international criteria (not based upon MRI findings), including the guidelines of the National Institute on Aging-Alzheimer’s Association for probable AD dementia (McKhann et al., 2011), the 2005 consensus criteria for probable DLB (McKeith et al., 2005), the diagnostic criteria of SD (Gorno-Tempini et al., 2011), and the PCA criteria proposed by Tang-Wai (Tang-Wai et al., 2004).

### Neuropsychological Assessment

A full set of standardized neuropsychological scales was used to evaluate cognitive function, including global function, memory, language, attention, executive function, visuospatial abilities, and other non-cognitive domains. Subjects were tested through the following instruments: Mini-Mental Status Examination (MMSE) (Katzman et al., 1988), global function; Memory and Executive Screening test (MES) (Guo et al., 2012), immediate and delayed recall abilities; Auditory Verbal Learning Test (AVLT) (Guo et al., 2009), episodic memory; Boston Naming Test (BNT) (Guo et al., 1991), visual naming ability; Digit Symbol Substitution Test (DSST) (Wechsler, 2008), attention; Shape Trail Test (STT) (Zhao et al., 2013), executive function; Judgment of Line Orientation (JLO) test (Qualls et al., 2000), visuospatial perception; Rey-Osteriche Complex Figure Test (CFT) (Guo et al., 2009), memory and visuospatial abilities; Hamilton Anxiety Scale (HAMA) (Hamilton, 1959); and the Hamilton Depression Scale (HAMD) (Hamilton, 1960), anxiety, and depression. All the assessments were conducted by five trained examiners in Huashan Hospital, who knew nothing about the participants’ diagnoses.

### Neuroimaging Data Acquisition

Whole-brain structural MRI data was obtained on a 3.0 T Siemens scanner (Erlangen, Germany) at Huashan Hospital by using a sagittal magnetization-prepared rapid gradient echo (MPRAGE) three-dimensional T1-weighted imaging sequence with 1 mm^3^ isotropic resolution: repetition time (TR) = 2300 ms; echo time (TE) = 2.98 ms; flip angle = 9°; field of view (FOV) = 240 mm × 256 mm; matrix = 240 × 256; 192 slices; and slice thickness = 1.0 mm.

### Neuroimaging Processing

Image analysis was carried out using the FreeSurfer image analysis suite (version 5.3.0)^1^. Detailed procedures have been described in previous publications (Fischl et al., 2002; Ségonne et al., 2004). First, the entire hippocampus was initially segmented using the routine pipeline. In brief, the processing includes the correction for head motion, the removal of non-brain tissue utilizing a hybrid watershed/surface deformation algorithm, automated Talairach transformation, and segmentation of the subcortical and cortical structures (including hippocampus, amygdala, caudate, putamen, and ventricles) based on a probabilistic brain atlas.

Next, automated segmentation of the hippocampal subfields was conducted via a Bayesian modeling approach and a calculation model of the areas surrounding the hippocampus. This method relied on a previously built atlas mesh of the hippocampal formation, which was constructed from the manual delineations in ultra-high-resolution MRI scans of 10 individuals (Van Leemput et al., 2009). It was reported that the average Dice coefficient was approximately 0.7 between the automated and manual segmentation methods for all the subfields except the fimbria and the hippocampal fissure (around 0.5) (Van Leemput et al., 2009). More details about the borders used to define the hippocampal subfields have been published previously (Van Leemput et al., 2009).

**FIGURE 1:**
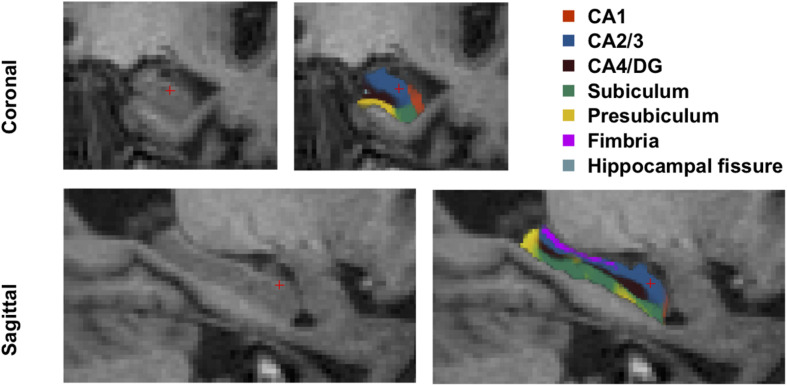
Hippocampal subfield segmentation. CA, cornu ammonis; DG, dentate gyrus.

Hence, the left and right hippocampi were automatically segmented into seven subfields: CA1, CA2/3, CA4/DG, subiculum, presubiculum, fimbria, and hippocampal fissure. The entire hippocampal volume was defined as the sum of the volume of all hippocampal subfields. We disregarded the fimbria and fissure in the following analysis since lack of accuracy of the segmentation of these two subfields has been reported (Van Leemput et al., 2009; Wisse et al., 2014). The hippocampal subfield segmentation results are illustrated in Figure 1.

The estimated total intracranial volume (eTIV) of each subject was also calculated using the standard FreeSurfer processing pipeline, which was used to correct for individual differences in head size in the subsequent statistical analysis.

### Statistical Analysis

Data were analyzed using SPSS (version 20.0; IBM Corp, Armonk, NY, United States) and Medcalc (version 11.4; Medcalc Software, bvba). The chi-square test was used to evaluate differences among categorical variables. Analysis of variance (ANOVA) was applied to evaluate differences among continuous variables. Correlation analyses were performed to examine the relationships between hippocampal subfield volumes and eTIV/age. No significant correlation was found. Therefore, comparisons of hippocampal subfield volumes between groups were examined with ANOVA and separately performed for the left and right hemispheres. Statistically significant differences based on ANOVA (*p* < 0.05) were further explored using *post hoc* pairwise Bonferroni tests. The receiver operating characteristic (ROC) curve was used to evaluate the ability of the volume of specific hippocampal subregions in identifying patients with a certain type of dementia. The method of comparing the areas under the curve (AUC) derived from Hanley JA (Hanley and McNeil, 1983). Spearman correlation analyses were applied between memory scores and hippocampal subfields. A chosen significance level was set at *P* < 0.05.

### Data Availability Statement

The data that support the findings of this study are available from the corresponding author upon reasonable request.

## Results

### Demographic and Clinical Information

The demographic and clinical data for all subjects are summarized in Table 1. The six groups were matched in terms of age, sex, and years of education. Between-group differences in the total score of MMSE (*p* < 0.05), MES (*p* < 0.05) were observed. As expected, all of these neuropsychological test scores were significantly lower in all patient groups than in the NC group (*p* < 0.05). The average MMSE and MES scores in each patient group were around 20 and 50, respectively, conforming to the inclusion criteria for mild dementia.

**TABLE 1:** Clinical data of tAD, LSD, RSD, DLB, PCA, and NC groups (mean ± SD).

	tAD (*n* = 22)	LSD (*n* = 17)	RSD (*n* = 17)	DLB (*n* = 15)	PCA (*n* = 12)	NC (*n* = 20)	*p*
Age	60.36 ± 3.75	61.71 ± 6.81	63.29 ± 6.90	60.13 ± 6.27	57.33 ± 6.36	61.00 ± 3.26	0.123
Sex (male/female)	12/10	9/8	8/9	9/6	6/6	9/11	–
Education (years)	12.64 ± 2.70	12.24 ± 2.75	11.35 ± 2.71	10.87 ± 2.39	10.67 ± 2.23	11.15 ± 2.39	0.154
Total score of MMSE	22.32 ± 2.85	20.71 ± 4.09	22.88 ± 3.72	20.7 ± 4.48	20.8 ± 3.74	28.10 ± 1.37	<0.05
Total score of MES	58.27 ± 13.32	52.06 ± 11.73	58.00 ± 12.56	54.93 ± 15.48	58.67 ± 9.68	82.85 ± 12.98	<0.05

*tAD, typical Alzheimer’s disease; LSD, left semantic dementia; RSD, right semantic dementia; DLB, dementia with Lewy bodies; PCA, posterior cortical atrophy; NC, normal control; SD, standard deviation; MMSE, mini mental status examination; MES, memory and executive screening test.*

### Comparison of the Left and Right Hippocampal Subregion Volumes of Each Group

We calculated and compared the left and right volumes of hippocampal subregions in all groups, respectively (Table 2). The results show that, in the NC group, the total volume of the left hippocampus was smaller than that of the right (*p* = 0.002), especially CA1 (*p* = 0.007), CA2/3 (*p* = 0.003), and CA4/DG (*p* = 0.008) although no significant difference was found in subiculum and presubiculum (*p* > 0.05). In the tAD group, the atrophy of the left hippocampus was more severe than its right counterpart (*p* = 0.032), mainly in CA1 (*p* < 0.001), CA2/3 (*p* = 0.008), and CA4/DG (*p* = 0.017). In the LSD group, the atrophy in the left was significantly more severe than that in the right (*p* < 0.001), containing all subregions. In the RSD group, atrophy in the right was significantly more severe than left (*p* = 0.002), including all subregions except CA1 (*p* = 0.421). In the DLB and PCA groups, no significant difference was found in the total volumes of the bilateral hippocampi as well as all subregions.

**TABLE 2:** Volumes of bilateral hippocampal subfields in all groups (mean ± SD, mm^3^).

NC	Left	Right	*t*	*p*
CA1	322.1 ± 41.1	343.3 ± 40.1	–3.023	0.007
CA2/3	950.2 ± 113.9	1016.7 ± 120.3	–3.425	0.003
CA4/DG	536.6 ± 65.7	569.8 ± 65.3	–2.955	0.008
Subiculum	625.28 ± 77.5	648.66 ± 58.6	–1.836	0.082
Presubiculum	444.19 ± 41.7	450.65 ± 45.7	–0.636	0.532
Total hippocampus	3328.3 ± 364.4	3520.8 ± 331.2	–3.698	0.002
tAD	*Left*	*Right*	*t*	*p*
CA1	295.2 ± 41.6	324.4 ± 44.3	–5.016	0.000
CA2/3	848.9 ± 133.3	910.9 ± 164.8	–2.914	0.008
CA4/DG	474.9 ± 72.0	505.2 ± 89.0	–2.583	0.017
Subiculum	523.01 ± 82.2	526.68 ± 78.6	–0.324	0.749
Presubiculum	375.94 ± 67.8	367.14 ± 73.0	0.773	0.448
Total hippocampus	2887.4 ± 398.2	3021.6 ± 467.2	–2.290	0.032
LSD	*Left*	*Right*	*t*	*p*
CA1	302.2 ± 58.6	329.6 ± 43.5	–2.286	0.036
CA2/3	684.1 ± 161.1	851.5 ± 164.1	–4.630	0.000
CA4/DG	386.9 ± 88.1	478.8 ± 91.1	–5.527	0.000
Subiculum	436.70 ± 96.8	564.04 ± 104.1	–7.149	0.000
Presubiculum	281.74 ± 66.3	379.12 ± 92.1	–5.833	0.000
Total hippocampus	2429.8 ± 494.9	3041.9 ± 541.5	–5.936	0.000
RSD	*Left*	*Right*	*t*	*p*
CA1	313.6 ± 51.9	300.8 ± 80.3	0.826	0.421
CA2/3	776.8 ± 144.8	696.9 ± 212.2	2.353	0.032
CA4/DG	434.1 ± 80.7	378.2 ± 115.9	3.265	0.005
Subiculum	503.73 ± 84.7	410.77 ± 119.3	5.155	0.000
Presubiculum	343.93 ± 74.5	283.47 ± 75.9	3.677	0.002
Total hippocampus	2770.5 ± 466.7	2409.3 ± 652.8	3.806	0.002
DLB	*Left*	*Right*	*t*	*p*
CA1	320.2 ± 40.3	337.8 ± 41.4	–1.701	0.111
CA2/3	891.0 ± 150.2	948.0 ± 142.9	–2.535	0.054
CA4/DG	507.6 ± 75.5	534.8 ± 69.9	–2.168	0.058
Subiculum	563.43 ± 61.0	570.56 ± 75.2	–0.492	0.631
Presubiculum	404.17 ± 55.2	391.95 ± 48.5	0.971	0.348
Total hippocampus	2887.4 ± 398.2	3021.6 ± 467.2	–1.845	0.086
PCA	*Left*	*Right*	*t*	*p*
CA1	309.3 ± 53.1	325.3 ± 50.6	–1.353	0.203
CA2/3	815.4 ± 148.6	813.6 ± 133.4	0.072	0.944
CA4/DG	462.0 ± 88.0	456.0 ± 76.1	0.456	0.657
Subiculum	529.38 ± 104.5	515.11 ± 89.5	1.064	0.310
Presubiculum	387.23 ± 76.1	358.59 ± 63.6	2.150	0.055
Total hippocampus	2882.3 ± 542.4	2859.1 ± 452.7	0.329	0.748

*tAD, typical Alzheimer’s disease; LSD, left semantic dementia; RSD, right semantic dementia; DLB, dementia with Lewy bodies; PCA, posterior cortical atrophy; NC, normal control; SD, standard deviation; CA, cornu ammonis; DG, dentate gyrus.*

### Comparison of Hippocampal Subfield Volumes of the Six Groups

We first analyzed the volumes of the left hippocampal subfields of all groups. In the LSD and RSD groups, all subfields were smaller compared with the NC group, except for left CA1 (*p* > 0.05). In the DLB group, the left hippocampus tended to be preserved compared to the NC group, including all subfields (*p* > 0.05). However, no significant difference was detected between the tAD and PCA groups (*p* > 0.05).

Then, we analyzed the volumes of the right hippocampal subfields of the six groups. We found a significant decrease in RSD and PCA patients compared with NC subjects in all subfields except for right CA1 (*p* > 0.05). Interestingly, we observed no significant difference between the tAD, LSD, DLB, and PCA groups (*p* > 0.05).

**FIGURE 2:**
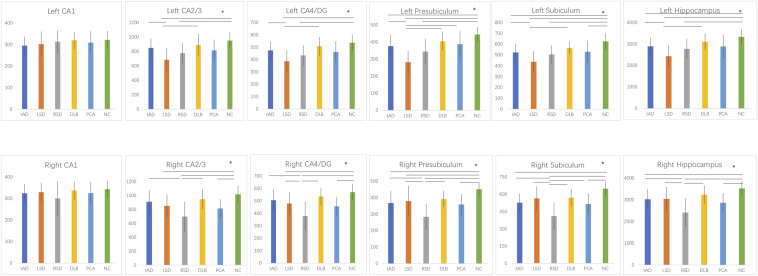
Between-group comparisons of hippocampal subfield volumes (*, Bonferroni-corrected *p* < 0.05). tAD, typical Alzheimer’s disease; LSD, left semantic dementia; RSD, right semantic dementia; DLB, dementia with Lewy bodies; PCA, posterior cortical atrophy; NC, normal control; CA, cornu ammonis; DG, dentate gyrus.

The volumetric differences between the groups are illustrated in Figure 2.

### Evaluation of Diagnostic Accuracy

ROC curve analysis was then performed to assess the ability of each hippocampal subregion to distinguish one group from the others. We differentiated one specific patient group from all other subjects by pulling all groups together. AUCs of the global hippocampus versus subregion volumes were then compared to test whether the measurement of the subregion was more accurate than the global hippocampus to discriminate between groups.

For the discrimination between tAD and other groups, only the AUC of the left CA1 was higher than 0.6 (0.624 [0.496–0.752]), better than the left hippocampus (0.542 [0.420–0.663]) and the right hippocampus (0.552 [0.425–0.678]).

To distinguish LSD, the AUCs of all subfields were higher than 0.5, and the left presubiculum (mean [95% CI] = 0.878 [0.785–0.972]) performed better than the whole left hippocampus (0.810 [0.694–0.925]) and the whole right hippocampus (0.472 [0.336–0.608]).

To distinguish RSD, all AUCs were higher than 0.5 except for CA1 (0.470 [0.319–0.621]), and the performance of the right CA4/DG (0.870 [0.754–0.986]), the right presubiculum (0.869 [0.758–0.980]), and the right subiculum (0.868 [0.765–0.971]) were better than the total right hippocampus (0.854 [0.740–0.967]) and left hippocampus (0.598 [0.467–0.730]).

To distinguish DLB, the AUCs of all subfields were higher than 0.5. The atrophy of the right CA4/DG (0.661 [0.536–0.785]), the left CA4/DG (0.642 [0.496–0.787]), and the right CA2/3 (0.629 [0.496–0.761]) could identify DLB better than the left hippocampus (0.620 [0.478–0.761]) and the right hippocampus (0.619 [0.485–0.753]).

To distinguish PCA, the AUCs of the right CA2/3 (0.658 [0.506–0.809]) and the right CA4/DG (0.646 [0.493–0.798]) were better than the right hippocampus (0.626 [0.476–0.776]) and the left hippocampus (0.506 [0.338–0.675]).

**FIGURE 3:**
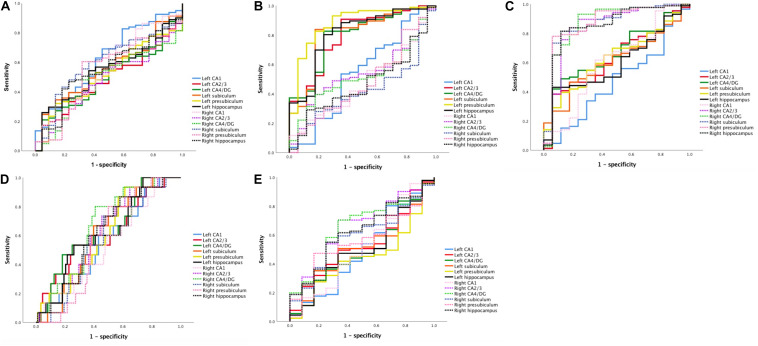
ROC curves for classification using hippocampal subfield measures. **(A)** ROC curve for tAD classification. **(B)** ROC curve for LSD classification. **(C)** ROC curve for RSD classification. **(D)** ROC curve for DLB classification. **(E)** ROC curve for PCA classification. ROC, receiver operating characteristic; tAD, typical Alzheimer’s disease; LSD, left semantic dementia; RSD, right semantic dementia; DLB, dementia with Lewy bodies; PCA, posterior cortical atrophy; CA, cornu ammonis; DG, dentate gyrus.

However, although the AUCs of some subfields were larger than the total hippocampus, none of them observed any significant difference (*p* > 0.05). The results are demonstrated in Figure 3 and Table 3.

**TABLE 3:** Optimal indicators (AUC > 0.6) and their differential efficacy of the hippocampal subregions in patients with tAD, LSD, RSD, DLB, and PCA.

Group	Subregion	Volume (mm^3^)	AUC	Sensitivity	Specificity
tAD	Left CA1	≤275.6	0.624*	0.827	0.409
LSD	Left presubiculum	≤326.5	0.878*	0.826	0.824
	Left hippocampus	≤2648.5	0.810	0.802	0.765
	Left subiculum	≤475.9	0,809	0.837	0.824
	Left CA2/3	≤701.3	0.794	0.907	0.647
	Left CA4/DG	≤416.8	0.794	0.826	0.765
RSD	Right CA4/DG	≤411.5	0.870*	0.930	0.765
	Right presubiculum	≤326.1	0.869*	0.779	0.941
	Right subiculum	≤486.3	0.868*	0.814	0.882
	Right hippocampus	≤2758.2	0.854	0.814	0.882
	Right CA2/3	≤769.6	0.846	0.837	0.824
	Left CA4/DG	≤477.8	0.657	0.547	0.765
	Left presubiculum	≤358.7	0.633	0.651	0.588
	Left CA2/3	≤808.2	0.629	0.593	0.588
	Left subiculum	≤536.7	0.609	0.581	0.647
	Right CA1	≤323.7	0.603	0.616	0.647
DLB	Right CA4/DG	≥501.3	0.661*	0.800	0.591
	Left CA4/DG	≥520.3	0.642*	0.533	0.750
	Right CA2/3	≥877.4	0.629*	0.733	0.545
	Left hippocampus	≥3159.4	0.620	0.533	0.739
	Right hippocampus	≥2993.6	0.619	0.733	0.523
	Left presubiculum	≥386.3	0.616	0.600	0.523
	Left CA2/3	≥931.1	0.614	0.533	0.750
	Left subiculum	≥555.4	0.605	0.667	0.602
PCA	Right CA2/3	≤808.2	0.658*	0.703	0.667
	Right CA4/DG	≤453.4	0.646*	0.703	0.667
	Right hippocampus	≤2993.6	0.626	0.549	0.750

*, the AUC of the subregion was higher than that of the total hippocampus. Comparison of the AUCs between subfield and the total hippocampus showed no significant difference (*p* > 0.05). AUC, areas under the curve; tAD, typical Alzheimer’s disease; LSD, left semantic dementia; RSD, right semantic dementia; DLB, dementia with Lewy bodies; PCA, posterior cortical atrophy; CA, cornu ammonis; DG, dentate gyrus.

### Associations Between Hippocampal Subfields and Memory Recall Scores

Table 4 lists the Spearman correlation analysis results. Patients with SD, PCA, and DLB were not included because of their relative retention of episodic memory. There were significant correlations between bilateral whole hippocampal volume and immediate and delayed recall scores (*p* < 0.05). Furthermore, the results showed that regarding three times immediate recall scores, there were positive correlations with the left CA1, CA2/3, CA4/DG, subiculum, and presubiculum (*p* < 0.05). In addition, delayed recall scores were strongly and positively associated with the bilateral CA2/3, CA4/DG, subiculum, and presubiculum (*r* = 0.38–0.52, *p* < 0.05).

**TABLE 4:** Correlation analysis between hippocampal subfield volumes and Immediate recall and Delayed recall scores (*r*, *p*).

Subfields	MES IR1	MES IR2	MES IR3	MES DR
Left CA1	0.222, 0.193	0.343, 0.041*	0.124, 0.473	0.260,0.126
Left CA2/3	0.304, 0.015*	0.315, 0.012*	0.331, 0.049*	0.430, 0.009*
Left CA4/DG	0.334, 0.008*	0.306, 0.014*	0.342, 0.041*	0.464, 0.004*
Left subiculum	0.320, 0.011*	0.325, 0.010*	0.312, 0.064	0.521, 0.001*
Left presubiculum	0.374, 0.024*	0.357, 0.032*	0.291, 0.085	0.502, 0.002*
Right CA1	0.129, 0.453	0.230, 0.177	0.139, 0.420	0.289, 0.088
Right CA2/3	0.337, 0.055	0.256, 0.131	0.196, 0.252	0.499, 0.002*
Right CA4/DG	0.167, 0.124	0.289, 0.087	0.217, 0.203	0.474, 0.003*
Right subiculum	0.392, 0.068	0.342, 0.072	0.291, 0.085	0.380, 0.000*
Right presubiculum	0.238, 0.161	0.195, 0.256	0.175, 0.307	0.447, 0.006*
Left hippocampus	0.422, 0.010*	0.419, 0.011*	0.288, 0.089	0.517, 0.001*
Right hippocampus	0.379, 0.023*	0.354, 0.034*	0.140, 0.198	0.560, 0.000*

r, correlation coefficient. *, *P* < 0.05. IR, immediate recall; DR, delayed recall; CA, cornu ammonis; DG, dentate gyrus.

## Discussion

Discrimination between patients with different causes of dementia at early stages is quite challenging as the clinical patterns of cognitive impairment may not yet be fully established. This is the first study to segment the hippocampus into four types of dementia, namely tAD, SD (including LSD and RSD), DLB, and PCA, to explore different atrophy patterns and help with diagnosis. This automated technique is publicly available, reproducible, and has been validated against manual volume estimations (Van Leemput et al., 2009).

First, we compared the volume of bilateral hippocampal subregions in dementia patients and normal populations. In both NC and tAD patients, the volume of the left hippocampus was smaller than right, mainly in the CA1, CA2/3, and CA4/DG subregions. In LSD patients, the volume of the left hippocampus was smaller than that of the right, including all subregions, and in RSD patients, the atrophy of the right hippocampus was more severe than the left. There was no significant difference in the volume of bilateral hippocampus in DLB and PCA patients, including all hippocampal subregions, indicating that the hippocampal volume of the two was relatively symmetrical.

Then, the volumetric comparisons between groups were performed. The results addressed in particular (Mu and Gage, 2011) tAD, LSD, RSD, and PCA patients had widespread subregional atrophy in bilateral hippocampi compared to NC while the volume of the bilateral hippocampi and subfields tended to be preserved in DLB patients; (Hatanpaa et al., 2014) among all groups, atrophy of the left hippocampal subfields in the LSD group was the most serious of all, and RSD patients were the most severely atrophied in the right hippocampus; (Freundl and Buzsi, 2015) no significant difference was found in the volume of subregions between tAD and PCA patients. These findings may serve the differential diagnosis.

ROC analysis was further conducted and revealed that some hippocampal subfield measurements were more accurate than the global hippocampus in discrimination, but none observed significant difference. Moreover, relation analysis revealed that several hippocampal subfields were significantly related to memory recall scores.

Among all groups, the volume decreases of the left hippocampus were maximal in LSD patients, including left CA2/3, CA4/DG, subiculum, and presubiclum although, in RSD patients, the predominance of atrophy in the right hippocampus, including right CA2/3, CA4/DG, subiculum, and presubiclum, were found compared to all the other groups. Previous studies reported a stronger hemispheric and anterior–posterior asymmetry of hippocampal atrophy in SD compared with AD (Chan et al., 2001; Galton et al., 2001; La Joie et al., 2013). However, they have always mixed left and right SD for comparison, and to our knowledge, this is the first study to divide SD patients into left and right subgroups for hippocampal subregion analysis, and therefore, it may be more accurate for further comparisons.

Researchers have suggested that, in DLB patients, episodic memory impairment may not occur in the early stages compared to AD (Calderon et al., 2001; McKeith et al., 2005). In our study, the DLB group showed no significant atrophy of all hippocampal subregions compared to NC, and we failed to show statistically significant differences between the DLB and tAD groups. Similarly, one previous study also indicated that AD and DLB did not reveal significant differences, but AD exhibited significantly greater atrophy in CA1, CA2/3, and subiculum bilaterally while DLB showed left-predominant atrophy in CA1 and subiculum compared to NC (Chow et al., 2012). Other studies found that DLB patients showed less atrophy of CA1 and subiculum in comparison to AD (Firbank et al., 2010; Mak et al., 2016). Researchers also reported that the hippocampal atrophy in DLB was less severe than in AD (Elder et al., 2017; Mak et al., 2017). Although these findings seem to be various, there are several plausible explanations. First, DLB is a heterogeneous disease, which exhibits coexisting AD pathology, such as amyloid plaques and neurofibrillary tangles. The hippocampal atrophy might be similar between AD and DLB groups. Second, it has been proposed from autopsy studies that tau may first affect the boundary of subregions (Lace et al., 2009), which stresses the importance of delineating the boundaries during segmentation. As the definitions of the hippocampal subfield boundaries obtained using FreeSurfer may vary from other techniques, the results in our study should be interpreted with caution.

In this study, tAD and PCA patients did not differ in terms of subfield volumetry. Although the absence of a significant difference could be due to a lack of statistical power, there are still several possible reasons. First, since the most common pathological changes of PCA patients are senile plaque deposition and nerve fiber tangles located in the posterior cortex of the brain, researchers generally believe that PCA is attributable to AD in the majority of patients (Crutch et al., 2017). Literatures have reported that the hippocampal atrophy in AD patients with early onset is relatively mild compared to patients with late onset (Shiino et al., 2006; Frisoni et al., 2007). This may generate a further narrowing of the differences between the two. Second, although it has been reported that PCA patients can have atrophy of the medial temporal lobe, including the hippocampus, there is no unified conclusion. Some studies report that the volume of gray matter in the left medial temporal lobe or the hippocampus of PCA patients was larger than that in AD patients (Kas et al., 2011; Wang et al., 2015). However, other studies find no significant difference in the volume of bilateral hippocampus and medial temporal lobes of PCA and tAD (Lehmann et al., 2011). On the other hand, the asymmetrical hippocampal atrophy was greater in tAD patients than in PCA patients, suggesting that tAD subjects were more vulnerable to unilateral atrophy, which may help diagnosis in clinic work.

In this study, we observed that hippocampal subfield volumes were closely correlated with memory abilities with a stronger relationship between left hippocampal subfield volumes and delayed recall scores. One study found positive correlations between the presubiculum and subiculum volumes and delayed recall scores in the AD group, which is consistent with our results (Lim et al., 2013). Another study analyzed the correlation between the hippocampus and episodic memory tests in 133 normal individuals, indicating that CA1 and subiculum were essential in episodic memory (Zammit et al., 2017). Other studies report that CA1 was strongly involved in memory performance (Adamowicz et al., 2017; Comper et al., 2017). However, we did not find a significant correlation between the volume of CA1 and memory recall scores. One possible reason is that, due to the placement of the subfield boundaries in FreeSurfer, a large portion of the subfields may be allocated to adjacent subfields, which leads to volume estimates that disagree with anatomical studies (Wisse et al., 2014). For instance, large parts of CA1 may be assigned to subiculum and CA2/3. We observed no significant difference in CA1 between groups, and it was the same as previous studies using Freesurfer (Hanseeuw et al., 2011; Li et al., 2016) but in contrast with the findings adopting other techniques. However, significant hippocampal neuronal cell loss in CA1 and the subiculum is typical in AD (West et al., 1994, 2004), and CA1 appears to be related to the severity of AD pathology in previous neuropathologic studies (Giannakopoulos et al., 2009). Therefore, the interpretation of the CA1 results in our study should be cautious.

Previous studies mainly focus on the overall structure or function of the hippocampus, and only a few chose to analyze at the subregional level. This research explored the bilateral hippocampal subfield atrophy pattern and compared the efficiency of the different hippocampal subregions in the identification of four types of mild dementia. The major strengths of this study include the multiple types of dementia (especially dividing SD into left and right groups), the state-of-the-art imaging analysis, and its focus on the mild dementia stages. However, several limitations must be considered. First, due to the low incidence of some types of dementia, the sample size included in this study is relatively small. Although age, gender, and education were matched in this study to control the variation between groups, imbalances caused by other factors could still be present. Second, since this study is cross-sectional, there is a lack of observation on the dynamic changes of hippocampal subfield atrophy throughout the disease course. Some hippocampal subregions may not shrink significantly at the stage of mild dementia, but different atrophy patterns may occur as the disease progresses, which was not followed up in this study. Third, although all the patients met the clinical diagnostic criteria, there was no pathological evidence to further support the diagnosis. Moreover, it should be noted that the subfield volumetry analysis only provides approximations of the hippocampal subfield volumes based on anatomical landmarks derived from atlases. More focus needs to be addressed toward the standardization of acquisition and analysis methods to facilitate the integration of findings across studies.

In addition, the functional connections of diverse hippocampal subregions and their relationship with cognition may also play an important role in the onset and progression of dementia. Therefore, we intend in the future to combine the functional imaging, cerebral metabolism with cognitive function and study longitudinal changes in hippocampal subfield measures to enrich our understanding of the hippocampal network and establish a new basis for disease diagnosis and prediction. A longer follow up in a larger sample that could examine the distinct changes in the hippocampal subfield atrophy patterns would be of future interest to validate our findings in this study.

## Conclusion

Using an automated image analysis pipeline to explore the subfields of the hippocampus, our study reveals that patients with tAD, LSD, RSD, DLB, and PCA have different atrophy patterns in bilateral hippocampi at mild stages of dementia. The atrophy of left CA1 helped differentiate tAD; the left presubiculum was most atrophied in LSD; the volume of right CA4/DG, right presubiculum, and right subiculum could identify RSD; the preservation of CA4/DG and right CA2/3 could distinguish DLB; the right CA2/3 and right CA4/DG helped to discriminate PCA. We also explored a significant correlation between hippocampal subfield volumes and delayed memory recall scores. These findings help to distinguish between patients with different types of dementia more effectively and indicate that the changes in hippocampal subfield volumes might be regarded as biomarkers of memory decline.

## Data Availability Statement

The raw data supporting the conclusions of this article will be made available by the authors, without undue reservation.

## Ethics Statement

The studies involving human participants were reviewed and approved by the ethics committee of Huashan Hospital. The patients/participants provided their written informed consent to participate in this study.

## Author Contributions

LH: analysis and interpretation of data and drafting the manuscript. KC: acquisition of data. XH: further analysis and revising the manuscript. QG: conception and design, acquisition of data, and revising the manuscript. All authors contributed to the article and approved the submitted version.

## Conflict of Interest

The authors declare that the research was conducted in the absence of any commercial or financial relationships that could be construed as a potential conflict of interest.

## Footnotes

1 http://freesurfer.net/
